# Descriptive Study of Language and Communication Disorders Using a Psychopathological Protocol in a Matched Control Group to a Sample of People with Mild Cognitive Impairment

**DOI:** 10.1192/j.eurpsy.2025.1738

**Published:** 2025-08-26

**Authors:** A. Moreno-Romero, A. Arjona-Valladares, N. Jimeno

**Affiliations:** 1 University of Valladolid, Valladolid, Spain; 2 Psychiatry, University of Valladolid, Valladolid, Spain

## Abstract

**Introduction:**

Aging leads to a progressive deterioration at the communicative level. The identification of language impairment in older adults could help to prevent or slow down the development of a possible neurocognitive disorder.

**Objectives:**

To evaluate psychopathological manifestations in language and communication by means of a psychopathological evaluation protocol in a control group of subjects matched by age and sex to a group of people with mild cognitive impairment.

**Methods:**

The sample consists of twenty healthy older adults (75% female, 25% male) with mean age of 84.15 years (SD = 6.81). A descriptive and observational study was carried out. Subjects of both sexes between 70 and 95 years of age, with absence of possible cognitive impairment, were included. The *Mini-Cognitive Examination* was used to assess cognitive performance, the *PRESEEA* interview was used to obtain the speech sample and a psychopathological assessment protocol.

**Results:**

Increasing age is associated with greater intensity of language impairment (R^2^ = .02, *p* = .047). In the MEC-35 total score, the control group shows a significantly higher performance than the patient group (F = 49.11, *p* < .001). A negative correlation appears between the total score of psychopathological manifestations and the variables ‘educational level’ (R^2^ = .23, *p* = .029) and ‘socioeconomic level’ (R^2^ = .33, *p* = .007).

**Conclusions:**

Anomia, perseverations, disintegrated language, concretism and paragrammatism are possible early indicators of cognitive impairment. The elaboration and application of both assessment protocols and speech therapy intervention programs in older adults may improve communication skills.

**Disclosure of Interest:**

None Declared

